# Reward network dysfunction is associated with cognitive impairment after stroke

**DOI:** 10.1016/j.nicl.2023.103446

**Published:** 2023-05-31

**Authors:** Franziska Wagner, Jenny Rogenz, Laura Opitz, Johanna Maas, Alexander Schmidt, Stefan Brodoehl, Markus Ullsperger, Carsten M. Klingner

**Affiliations:** a Department of Neurology, 07747 Jena University Hospital, Friedrich Schiller University Jena, Germany; b Biomagnetic Centre, 07747 Jena University Hospital, Friedrich Schiller University Jena, Germany; c Faculty of Natural Sciences, Institute of Psychology, 39106 Magdeburg, Germany; d Center for Behavioral Brain Sciences, Magdeburg, Otto-von-Guericke University Magdeburg, Germany

**Keywords:** Stroke, Reward network, MEG, Functional connectivity, CCN

## Abstract

Stroke survivors not only suffer from severe motor, speech and neurocognitive deficits, but in many cases also from a “lack of pleasure” and a reduced motivational level. Especially apathy and anhedonic symptoms can be linked to a dysfunction of the reward system. Rewards are considered as important co-factor for learning, so the question arises as to why and how this affects the rehabilitation of stroke patients.

We investigated reward behaviour, learning ability and brain network connectivity in acute (3-7d) mild to moderate stroke patients (n = 28) and age-matched healthy controls (n = 26). Reward system activity was assessed using the Monetary Incentive Delay task (MID) during magnetoencephalography (MEG). Coherence analyses were used to demonstrate reward effects on brain functional network connectivity.

The MID-task showed that stroke survivors had lower reward sensitivity and required greater monetary incentives to improve performance and showed deficits in learning improvement. MEG-analyses showed a reduced network connectivity in frontal and temporoparietal regions. All three effects (reduced reward sensitivity, reduced learning ability and altered cerebral connectivity) were found to be closely related and differed strongly from the healthy group.

Our results reinforce the notion that acute stroke induces reward network dysfunction, leading to functional impairment of behavioural systems. These findings are representative of a general pattern in mild strokes and are independent of the specific lesion localisation. For stroke rehabilitation, these results represent an important point to identify the reduced learning capacity after stroke and to implement individualised recovery exercises accordingly.

## Graphical abstract

**Figure:**
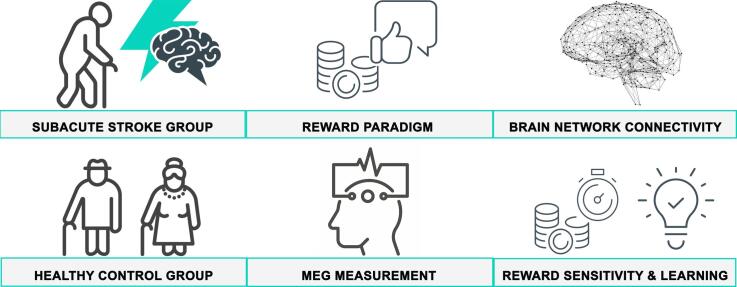

## 1 Introduction

Stroke represents the leading cause of long-term disability worldwide (Atteih et al., 2015, Benjamin et al., 2019, Ghose et al., 2005, Virani et al., 2020). The burden of disability among elderly stroke survivors is substantial (Kelly-Hayes et al., 2003). Not only motor and speech problems, but also manifest cognitive deficits are among the major limitations following stroke (Bour et al., 2011).

A crucial factor for effective rehabilitation outcome of stroke patients is the preserved motor learning ability (Dahms et al., 2020, Kitago and Krakauer, 2013, Krakauer, 2006, Lam et al., 2016). Several studies have shown that learning tasks performed under reward conditions substantially improve motor learning adaptation in stroke patients (Lam et al., 2016, Quattrocchi et al., 2017, Subramanian et al., 2010, van Vliet and Wulf, 2006).

In addition, about one third of stroke survivors suffer from apathy symptoms resulting in a reduced motivational level and a quantitative reduction in goal-directed behaviours. Anhedonia, on the other hand, refers to the inability to feel pleasure or loss of interest in previously rewarding activities (Berridge and Kringelbach, 2015, Husain and Roiser, 2018, Tay et al., 2021). Both apathy and anhedonia are a potential manifestation of reward system dysfunction post stroke and are further associated with cognitive deficits and can negatively influence rehabilitation outcome (Ayerbe et al., 2013, Mayo et al., 2009, Tay et al., 2021).

Seeking rewards is based on internalized innate mechanisms positively reinforcing goal-directed behaviour such as homeostasis, survival, and reproduction (Berridge and Kringelbach, 2015, Fellows, 2004, Haber and Knutson, 2010, O'Doherty et al., 2017, Schultz, 2000, Schultz, 2015). The core of the reward system is the mesocorticolimbic dopamine system comprising projections of midbrain dopamine neurons that protrude to regions of the prefrontal cortex, such as the medial prefrontal and orbitofrontal cortex (Haber and Knutson, 2010, Ikemoto, 2010, Lammel et al., 2008, Wise, 2002). From a functional viewpoint, the brain regions involved in the reward system are highly interconnected and include cortico-striatal loops that form a complex reward circuitry (Haber, 2016, Haber and Knutson, 2010).

Previous studies of stroke patients have focused primarily on lesion localization by lesion-symptom mapping. These studies have found an association between stroke lesions in key anatomical structures of the reward system and post stroke depression as also clinically apparent apathy (Narushima et al., 2003, Rochat et al., 2013). In contrast, other studies have shown a high number of stroke patients suffering from post-stroke depression or post-stroke apathy presenting without a clear association to the location of the stroke lesion (Carson et al., 2000, Kutlubaev and Hackett, 2014, Pan et al., 2022, Shi et al., 2017, Widmer et al., 2019).

The current understanding of brain function highlights behavioural functional deficits not a consequence of single structural lesions rather they are due to complex changes in brain network dynamics (Bonkhoff et al., 2021, Jaywant et al., 2022, Marsh et al., 2020, Tay et al., 2020). We herein propose that anhedonia and apathy syndromes after stroke are caused by disruptions in brain networks, particularly in frontal brain regions (Jorge et al., 2010, Marsh et al., 2020, Pan et al., 2022, Tay et al., 2020).

The orbitofrontal and prefrontal cortex in particular constitute key regions generating reward response. These frontoparietal brain regions are part of the cognitive control network (CCN) and the related default mode network (DMN) (Botvinick and Braver, 2015, Chau et al., 2018, Pessoa and Engelmann, 2010). Regions of cognitive control interact with the cognition-related DMN comprising temporoparietal regions in addition to frontoparietal areas (Corbetta and Shulman, 2002, Parro et al., 2018, Raichle et al., 2001, Spaniol et al., 2015, Vincent et al., 2008). Brain regions involved in the DMN and the CCN constitute a cross-over point between the reward system and cognitive networks during process and outcome simulations in goal-directed behaviours (Gerlach et al., 2014). To investigate cognitive brain network dynamics, connectivity models of magnetoencephalography data allow real time analyses with high temporal resolution (Tewarie et al., 2019). Those processes of cognitive control and decision making have been demonstrated to synchronized at lateral prefrontal theta band (4–7 Hz) oscillations (Cohen, 2011, Cohen and Donner, 2013, Duprez et al., 2020, Luft et al., 2013, Ullsperger et al., 2014). This is the first study that examines post-stroke reward network integrity and behavioural reward response using the reward paradigm Monetary Incentive Delay Task (MID) in subacute stroke patients recorded by MEG (Knutson et al., 2000).

Reward system integrity was investigated at two different levels *(i, ii)*. At the behavioural level *(i)*, reaction time responses following reward cues and performance improvements over three separated blocks were compared between the subacute stroke group and an age-matched healthy control group. For evaluating brain network integrity during reward response *(ii)*, functional connectivity analyses of frontal and temporoparietal brain regions were applied using coherence analyses of MEG data.

## 2 Materials and methods

### 2.1 Participants

The study was approved the ethics committee of University Hospital Jena (REST 2019-1473). All participants gave their written informed consent according to the Declaration of Helsinki*.* 54 participants without any dementia or psychiatric disease comprising 28 patients in the stroke group and 26 persons in the age-matched healthy control group were included in this study (Table 1).

**Table 1:** Overview of the Assessment in stroke group and healthy control group.

	Stroke Group		Control Group		
	Mean	STD	Mean	STD	*P*
*n*	28	–	26	–	–
Age	70.32	9.23	69.35	5.64	0.44
Sex female	13	–	14	–	–
MoCA	22.86	3.29	25.73	1.99	0.00*
BDI-II	6.89	5.67	4.88	4.34	0.21
SF-36: Physical functioning	45.00	37.07	83.08	18.23	0.00*
SF-36: Emotional well-being	76.71	15.74	82.31	11.55	0.197
SF-36: General health	49.29	17.99	64.04	11.23	0.001*
EQ5D	56.61	19.77	80.19	13.30	0.00*
Handedness Right total	22	–	25	–	–
Handedness Both total	6	–	1	–	–
Fugl-Meyer Left	56.18	18.7	66.00	0.00	0.00*
Fugl-Meyer Right	59.86	11.96	66.00	0.00	0.00*

*P < 0.05 Mann-Whitney-U.

**Fig. 1:**
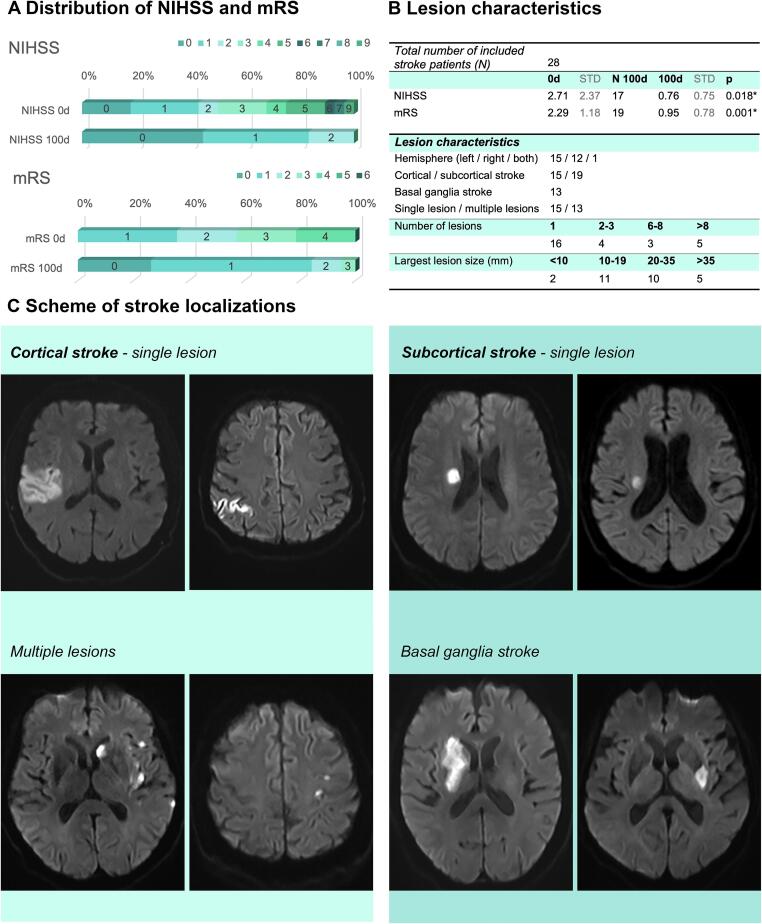
Characteristics of stroke patients included in the study. (A) Distribution of NIHSS and mRS scores in the subacute and the chronic phase after stroke indicated by stacked bar charts. (B) Lesion characteristics and clinical outcome after stroke (* indicates significant changes). (C) Representative clinical MRI scans of stroke patients included in the study.

Stroke patients were recruited from the Stroke-Unit of the University Hospital Jena within seven days after the acute lesion was identified in MRI (for details see Fig. 1). Only patients with minor middle cerebral artery (MCA) infarction and a first-ever stroke event were included (for more detailed stroke characteristics, see Fig. 1B, 1C). Considering the mean NIHSS score of 2.71 and the mean mRS score of 2.29 in the stroke group, the participating patients were predominantly mildly or moderately impaired by the stroke (NIHSS ranging from 0 to 9, mean = 2.71, STD = 2.37) (Brott et al., 1989, Kasner, 2006), (mRS ranging from 1 to 4, mean = 2.29, STD = 1,18) (Meyer et al., 2002, Rankin, 1957) (Fig. 1B). Stroke patients with moderate or severe aphasia were excluded from the study to avoid decreased language comprehension as a confounding factor.

In both groups, the Montreal Cognitive Assessment (MoCA) was used for screening for cognitive impairment (Nasreddine et al., 2005). To exclude depressive symptoms, the BDI-II was used (Beck et al., 1996). Performance based motor deficits of both arms were tested by the Fugl-Meyer Assessment (Fugl-M) (Fugl-Meyer et al., 1975). To score current health-related quality of life, the standardized patient-reported Short Form Health Survey (SF-36) (Ware Jr, 1999) and EQ-5D Score (EuroQol, 1990) were administered (Table 1). To exclude former psychiatric diseases or addictive disorders, participants were asked to respond to questions on past psychiatric diseases, specifically depression or addictive disorders. These were set as exclusion criteria. Handedness was assessed by means of the Edinburgh Handedness Inventory (Oldfield, 1971). Right-handed (total 47) and both-handed (total 7) subjects were included in the study (Table 1).

Out of initial 30 participating patients in the stroke group and 28 participants in the control group, a total of four participants had to be completely excluded. Two subjects (one from each group) were excluded because of psychiatric diseases in the past. One participant from the stroke group was excluded due to chronic stroke lesions seen in the MRI. Another person in the healthy control group was excluded because of the >20 points score in the BDI-II depression scale (Beck et al., 1996). One participant in the control group was excluded only from the first block of the MID paradigm because of incorrect execution, while the other two blocks of the paradigm were included in the analyses.

The stroke group (28 S patients) and the control group (26 participants) in this study were age-matched (mean age stroke group 70.32, STD 9.23, mean age control group 69.35, STD 5.64, *P* = 0.44, ranging from 53 to 86, 27 females, Table 1). Age, handedness-score, and level of depression measured in the BDI-II were also comparable in both groups (Table 1). Considering the common cut-off value of 26 points, the dementia screening test (MoCA) was slightly reduced in both groups and differed significantly between the stroke and the control group (mean stroke group = 22.86, mean control group = 25.73, *P* = 0.00).

Physical functioning and general health measured using the SF-36, the subjective health status assessed by EQ5D, and the Fugl-Meyer Score detecting functional impairment of the upper extremity, differed significantly between the groups (Table 1).

### 2.2 Monetary Incentive Delay task

**Fig. 2:**
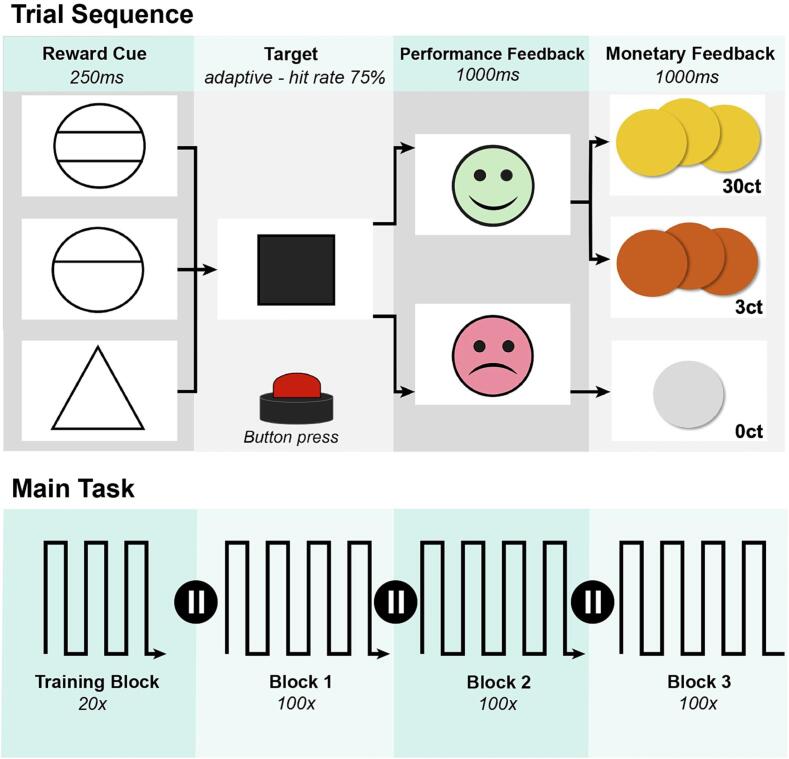
MID-Design Scheme of the Monetary Incentive Delay Task with block (B1-B3) design (Opitz et al., 2022). Every trial started randomly with one of three different reward incentive cues, lasting for 250 ms: a circle with one line indicated a possible gain of 3ct, a circle with two lines a gain of 30ct and a triangle meant no money could be won. Participants were told to hit the response button as fast as possible upon appearance of a white square (target) on the screen. Positive feedback is given by a green laughing smiley, otherwise a red sad smiley appeared. Second feedback with pictures of coins visualize the earned monetary rewards. Each Task consists of 300 trials equally divided into three blocks (B1, B2, B3). Target duration adapted to individual reaction times, resulting in a predestined hit rate of 75%. (For interpretation of the references to colour in this figure legend, the reader is referred to the web version of this article.)

The monetary incentive delay (MID) task is a well-established tool for studying reward system integrity (Knutson et al., 2000). The task employs visual cues coding for money incentives and requires the reaction of the participant to a target stimulus (Fig. 2). Our version consisted of 300 trials in three blocks (B1-B3) with about 100 trials each (MID design shown in Fig. 2). Before starting the paradigm, participants completed a training block comprising 20 trials. Individual understanding was ensured through active reproduction of the task instructions by the subject and checked in test runs preceding the measurement. Each trial randomly started with one of three different cues, lasting for 250 ms: a circle with one line indicated a possible gain of 3 cents, a circle with two lines a gain of 30 cents and a triangle meant no money could be won in the following trial. Participants were told to hit the response button as fast as possible upon appearance of a white square (target) on the screen. Between cue and target, a fixation cross with a randomized duration (750–1250 ms) was presented. If the button press did not exceed time limit, performance feedback was presented by a green laughing smiley, otherwise a red sad smiley appeared. Afterwards, pictures of coins visualized monetary rewards. The feedback was displayed for 1000 ms each. Target duration was adapted dynamically to individual reaction times, resulting in a predestined hit rate of 75%. Participants received a start budget of 30€ and could additionally earn about 20€. They were told at the beginning of the task that the money they earned would be paid as an expense allowance. Participants of the control group performed the MID task with their dominant hand, patients of the stroke group performed the task with their non-impaired hand regardless of the presence of a hemiparesis.

### 2.3 Statistical analysis of behavioural data

Mann-Whitney U Test was applied to analyse the distribution of data between the two groups using SPSS software (Version 27, IBM). To analyse behavioural reaction times, Generalized Estimating Equations (GEE) assuming a normal-distributed response (Halekoh et al., 2006, Liang and Zeger, 1986) were calculated using R (Version 4.1.1 / Kick Things) (R Core Team, 2020) and RStudio (Version 2021.09.0 Build 351) (RStudio Team, 2020). The goal of GEE, as an extension of generalized linear models (GLM), is to draw inferences from the population by accounting for the within-subject correlation of longitudinal data. Ignoring these correlations would lead to regression estimates being more widely scattered around the true population means. Reaction time outliers below 100 ms as well as all reaction times above 1000 ms were excluded to identify outliers. Limits were chosen to identify outliers that were either too short that they could not have occurred in response times or that they could no longer be confidently attributed to our stimulus as a direct response. Based on the resulting dataset, the mean and the standard deviation (STD) were calculated for each participant. For the final dataset, all participant-specific reaction times either below max (100, mean = -2.57*STD) or above mean = +2.57*STD were removed, roughly corresponding to the 99% confidence interval. In total, 5.6% of all not-missed reaction times were excluded. To analyse reaction times, we applied a 2x3x3 factorial design with the two-staged factor group (healthy, stroke) and the three-staged factors cue (0ct, 3ct, 30ct) and block (B1, B2, B3). Based on the three parameters cue, block, and group and their interaction terms, different parameter combinations as well as the two most reliable correlation structures (AR1, exchangeable) with different confounders were tested. Comparing multiple models, model M8 is the model of choice one of the lowest model selection criteria values and was used for further analyses (see Supplement Table 1).

### 2.4 MEG imaging

MEG imaging was undertaken in a magnetic shielded chamber in the Biomagnetic Centre of the University Hospital Jena. Data was acquired using an Elekta/Neuromag whole-head MEG system Vectorview 306. This MEG device comprises 306 channels, composed of 102 magnetometers and 204 planar gradiometers. A horizontal and a vertical EOG as well as a single ECG lead (second lead according to Einthoven) were recorded using Ag/AgCl electrodes. Furthermore, 4 HPI coils (head positioning indexing, 3 SPACE FASTRAK, Polhemus Inc. USA) were fixed to the test person’s head allowing for head localization in the MEG helmet during measurement. Coils and anatomical and functional landmarks on the test person’s head were digitized using 3D Fasttrak digitizer (Polhemus, inc. Colchester VT, USA). For stimulus presentation, we used the program Presentation (Neurobehavioral Systems, Inc. Berkeley, CA., USA, Version 16.3). The paradigm was shown on a screen in the chamber with a projector and participants could respond using an answer keyboard (LUMItouch photon control optical response pad).

### 2.5 Pre-processing of MEG data

**Fig. 3:**
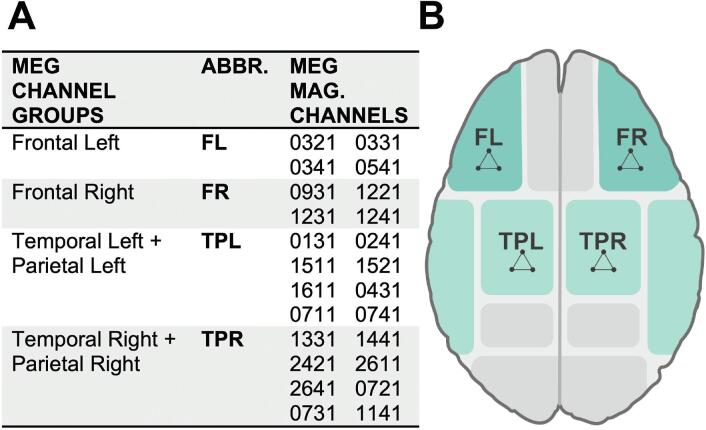
Selection of Channel groups and networks (A) MEG channel groups and corresponding MEG magnetometer channels (MAG.) (B) schematic representation of analysed brain region localizations (FL = frontal left; FR = frontal right; TPL = temporo-parietal left, TPR = temporo-parietal right).

MEG data were acquired using a sampling rate of 1 kHz. A bandpass-filter for 0,1–330 Hz was applied. For further raw data pre-processing, a Maxwell-filter (Software Version 2.2.10, Elekta Neuromag Oy. Finland) was used, removing interference signals from outside the MEG helmet (Taulu et al., 2005). Additionally, all data files were co-registered to one participant’s head position. Matlab fieldtrip toolbox (Oostenveld et al., 2011) was used to pre-process imaging data. The raw measurement files were segmented into trials lasting from 0.5 to 1.0 s around trigger onset. An automatic correction for jump artifacts was applied. Data were downsampled to 500 Hz and submitted to a visual artifact correction method known as reject visual, as implemented in the fieldtrip toolbox. Subsequently, an independent component analysis was performed to correct for eyeblink and heartbeat artifacts. In addition, other artifacts were also removed if these could be clearly identified. A bandpass-filter for 0.1–100 Hz was applied prior to subsequent analyses. MEG channels were merged into 10 groups corresponding to their underlying brain regions. For further analyses, we selected four frontal and temporoparietal channel groups (FL = Frontal Left; FR = Frontal Right; TPL = Temporoparietal Left; TPR = Temporoparietal Right; see Fig. 3 and Supplement Table 2). Coherence as functional connectivity tool is defined as the statistical relationship of oscillating neuronal signals considering phase synchrony and amplitude at a predefined frequency range (Bastos and Schoffelen, 2015, Bowyer, 2016, Friston, 2011, Kida et al., 2015). As a statistical approach, it allows to determine the degree to which two signals from different brain regions are correlated (Bowyer, 2016). As higher coherence values reflect increased neuronal interaction between brain regions, coherence serves as a measure of functional integration within neuronal networks (Srinivasan et al., 2007). Coherence was calculated with the fieldtrip toolbox (Oostenveld et al., 2011) employing a non-parametric approach event-related for frequencies of 1.0 to 40 Hz (in 1 Hz steps) for each channel combination. Data were Fast Fourier transformed (Cooley and Tukey, 1965) using DPSS (discrete prolate spheroidal sequences) as tapering function. Cross-spectral density (Appendix, Formula 1 (Kida et al., 2015)) was computed from frequency domain data. Lastly, using the cross-spectral density matrix, the coherence coefficient C_XY_(f) for signals X and Y representing pairwise channel group combinations was calculated (Appendix, Formula 2 (Kida et al., 2015)). The results were then submitted to statistical analyses.

### 2.6 Statistical analysis of MEG connectivity data

Coherence results were analysed using R (Version 4.1.1 / Kick Things) and RStudio (Version 2021.09.0 Build 351). Coherence coefficients of each channel combination were averaged separately for each frequency band. We were interested in group differences of the connectedness between prespecified channel groups (channel groups = networks). Coherence coefficients of each channel combination were calculated in the alpha 8–12 Hz, beta 13–30 Hz, gamma > 30 Hz, delta 0.5–3 Hz, theta 4–7 Hz) (Engel and Fries, 2010, Pereda et al., 2005). Only theta band coherence revealed significant results between groups. Inter-network connectivity was estimated applying coherence analyses between each combination of channels of the networks (Fig. 3). All connectivity values from all connections of interest were then averaged for each participant. Coherence values were then transformed to a z-score by means of Fisher’s z-transformation. This analysis was performed separately for each experimental condition, resulting in one averaged value per network-condition-subject. Within-group comparisons of the between network connectivity was performed by a paired *t*-test between the experimental conditions. To account for between-group differences, we used Welch’s two sample *t*-test by entering the connectivity difference between experimental conditions for each subject. All results were corrected for multiple comparison by using the false discovery rate (FDR).

### 2.7 Data availability

The datasets analysed during the current study are available from the corresponding author on reasonable request.

## 3 Results

### 3.1 Behavioural performance

**Fig. 4:**
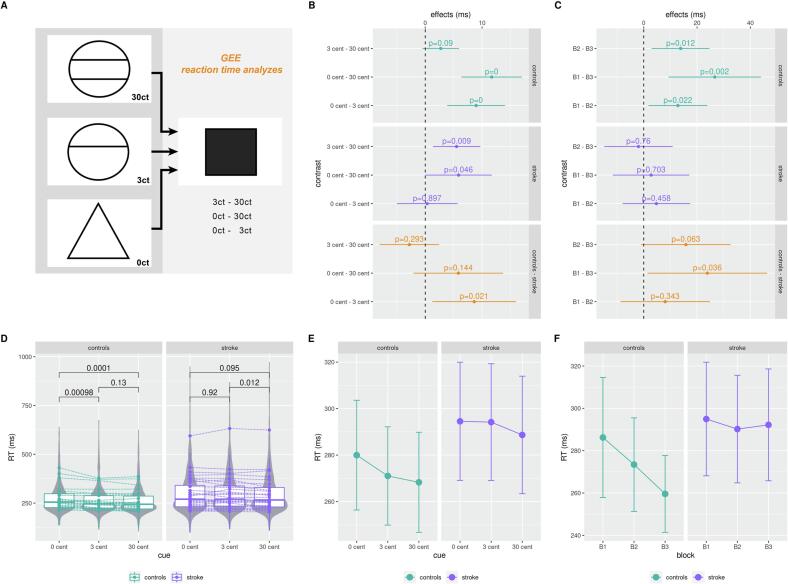
Analyses of behavioural reaction times (A) Scheme of the analysed MID-paradigm section (D) Boxplot: single mean reaction time data, lines/points correspond to different participants. *P*-values from paired Mann-Whitney U Test show significant reward-related speed of reaction time in the control group from 0ct to 3ct and 0ct to 30ct. Reward-related reaction time in stroke group did not improve significantly for low reward (between 0 vs 3ct), compared to high reward (0 vs 30ct). Violin plots are based on single reaction time data. (B, E) GEE model results for effects of group (stroke vs heathy) and cue (0ct, 3ct, 30ct) on reaction times. Reaction time differences between cues did not significantly differ for factor low reward compared to high reward (0 vs 30ct) in the stroke group. (C, F) Analysing the effect of the blocks on reaction times showed that the controls significantly improved performance from block B1 to B3. The stroke group showed no significant improvement between block B1 to B3. colour (code: green = healthy group, purple = stroke group, orange = contrast, B = block column). (For interpretation of the references to colour in this figure legend, the reader is referred to the web version of this article.)

Reaction times in the MID-Paradigm (Fig. 2) were analysed dependent on the factors group (stroke, healthy), expected reward (0ct, 3ct, 30ct), block (1, 2, 3), age, and MoCA score by using a GEE (generalized estimating equations) model (Fig. 4). We found a significant main effect of reward on reaction time for the high as well as the low reward cue (3ct, b = -8.95, stderr = 2.6, *P* < 0.001 and 30ct, b = -11.7, stderr 2.7, *P* < 0.001, Fig. 4). We also found a significant main effect of block on reaction time for blocks 2 and 3 (block 2, b = -12.81, stderr = 5.6, *P* = 0.02, block 3, b = -26.64, stderr = 8.8, *P* = 0.002). No significant main effect was found for the reaction time comparing stroke patients and healthy controls (b = 3.92, STD = 20.8, *P* = 0.85).

Most importantly, there was a significant interaction effect between low reward cue (3ct) and the subject group (stroke vs. healthy controls) (m = 8.61, stderr = 3.72, *P* = 0.021). No significant interaction effect was found between high reward cue (30ct) and subject group (m = 5.82, stderr = 4.0, *P* = 0.15). This indicates that for low reward differences (0vs.3ct), the amount of reward affected reaction times differently in stroke patients compared to healthy controls. This effect was limited to low reward differences while high reward differences (0vs.30ct) showed no difference in reaction times between stroke patients and healthy controls. In conclusion, reaction times were faster in high reward conditions for both stroke patients and healthy subjects without a significant effect of group.

To further investigate the effects of the factor expected reward and group on reaction times, we performed a post-hoc analysis (paired Mann-Whitney U Test) of the mean reaction time, dependent on the factors group (healthy, stroke) and expected reward (0ct, 3ct, 30ct) (Fig. 4D). There was a significant decrease in reaction time between cues 0ct and 3ct, as well as between 0ct and 30ct in the healthy group. The stroke group, on the other hand, only differed significantly between the 3ct and 30ct cue. Overall, participants in the stroke group showed a slower reaction time (RT) with a generally higher standard deviation compared to the healthy control group.

Additionally, we were further interested in the behavioural learning improvement over the blocks (factor block). For the factor block, we found a significant interaction effect for the third block and the subject group (m = 23.88, stderr = 11.4, *P* = 0.036), while no significant interaction effect emerged for the second block and the subject group (m = 8.06, stderr = 8.5, *P* = 0.34).

In conclusion, increasing practice duration of the MID paradigm (first block vs. third block) affects reaction times differently in stroke patients compared to healthy controls. Healthy controls exhibit greater improvement in reaction time between the first and the last block compared to stroke patients.

Furthermore, to investigate the effects of the factors block and group on reaction times, we performed a post-hoc analysis (paired Mann-Whitney U Test) of the mean reaction time dependent on the groups (controls, stroke) and block (1, 2, 3) (Fig. 4F). Decreasing mean reaction times over the three blocks were considered as preserved learning ability. In general, in the healthy group reaction, speed across all three blocks (B1-B2, B2-B3 and B1-B3) significantly improved and was associated with an overall improvement in positive behavioural learning. In contrast, the stroke group did not show any positive learning improvement during the entire experiment (Fig. 4C). In the direct group comparison using block contrasts, we detected group differences of reaction times over all blocks B1-B3 (m = 23.88, STD = 11.39, *P* = 0.036), but not for B1-B2 (m = 8.06, STD = 8.5, *P* = 0.343). Differences in the learning improvement between B2-B3 narrowly missed reaching significance (m = 15.82, STD = 8.5, *P* = 0.062), (Fig. 4C contrasts / 4F marginal means).

### 3.2 Functional connectivity to reward anticipation and the effects of stroke

We used a two-sample *t*-test to test for group differences in the reward anticipation cues (0ct, 3ct, 30ct) in the theta band (4–7 Hz). For each reward anticipation cue, we found significant group differences in connectivity analyses between the left frontal region and the left temporoparietal region and between the right frontal region and the right temporoparietal region (significant results are shown in Table 2).

**Table 2:** Significant group differences in MEG-Coherence during reward anticipation.^a^

Anticipated Reward	Region	Mean-Control	Mean-Stroke	t-value	df	r	*P*
0 ct	FL vs. TPL	0.29	0.2	3.28	38	0.47	0.002
0ct	FR vs. TPR	0.34	0.21	4.2	34	0.58	0.0002
3 ct	FL vs. TPL	0.28	0.2	3.1	42	0.43	0.003
3 ct	FR vs. TPR	0.32	0.23	3.2	34	0.48	0.003
30 ct	FL vs. TPL	0.28	0.19	3.1	38	0.46	0.003
30 ct	FR vs. TPR	0.33	0.23	3.7	34	0.53	0.0009

a Corrected for multiple comparisons by false discovery rate, analyses limited to the theta band (4–7 Hz).

### 3.3 Functional connectivity in graded reward anticipation

**Fig. 5:**
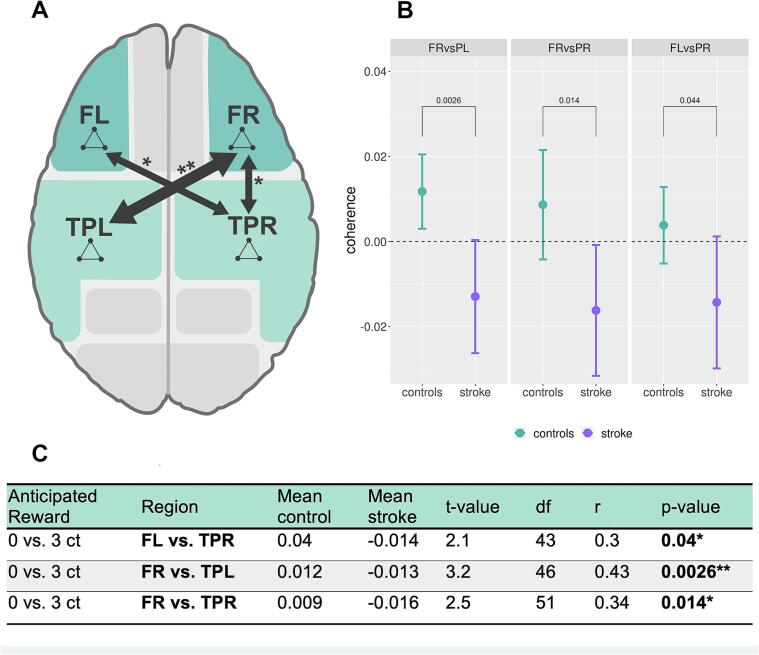
Channels group differences in coherence during reward anticipation 0ct vs. 3ct (A) Scheme of analysed brain regions. Arrows represent significant differences in coherence analyses between groups. (B) Boxplot of coherence data contrasting control condition and low reward anticipation (0ct vs 3ct) for FR vs TPL, FRs vs TPR and FL vs TPR with significant changes between groups stroke and control. (C) *t*-test for mean coherence value analyses between groups stroke and control in low reward prediction 0ct vs 3ct (Asterisks: one asterisk: *P* ≤ 0.05; two asterisks: *P* ≤ 0.01).

To test for group differences, we applied the contrast of theta band coherence between the incentive cue (3ct, 30ct) and the neutral cue (0ct) using Welch *t*-test between groups (contrasts used: 0vs.3ct, 0vs.30ct, 3vs.30ct in theta band coherence). Significant group differences appeared exclusively when the no-reward anticipation with the low reward anticipation (0vs.3ct) were compared. We found group differences in the functional connectivity (coherence) between the left frontal region and the right temporoparietal region (TPR), the right frontal region (FR) and the left temporoparietal region (TPL) as well as between the FR and the TPR (Table 3, Fig. 5).

**Table 3:** Group differences of MEG-Coherence contrasting differences in reward anticipation.^a^

Anticipated Reward	Region	Mean-Control	Mean-Stroke	t-value	df	r	*P*
0 vs. 3 ct	FL vs. TPR	0.04	−0.014	2.1	43	0.3	0.04
0 vs. 3 ct	FR vs. TPL	0.012	−0.013	3.2	46	0.43	0.0026*
0 vs. 3 ct	FR vs. TPR	0.009	−0.016	2.5	51	0.34	0.014*
0 vs. 30 ct	no significant group differences						
3 vs. 30 ct	no significant group differences						

a corrected by the false discovery rate, analyses limited to the theta band (4–7 Hz).

The control group exhibited higher reward-based modulation compared to the stroke group during anticipation of low reward cues in contrast to the control condition (mean 0.04 vs −0.014; *P*(FDR) = 0.04; df = 43). Comparing the FR and the TPR, the control group also showed higher reward-based modulation (mean 0.009 vs −0.016; *P*(FDR) = 0.014; df = 51). The largest group differences in reward-based modulation were detected between the FR and the TPL brain regions (mean 0.012 vs −0.013; *P*(FDR) = 0.0026; df = 43) when comparing the low reward with the control condition and between the control and the stroke group. The results are shown in Table 3 and Fig. 5. There were no significant results contrasting the high reward condition with the control condition during reward anticipation (0vs30ct) (Table 3, see Supplement Fig. 1).

### 3.4 The link between cerebral connectivity and behavioural performance in stroke patients

Our results show significant differences in the reward response between the stroke and control group to small reward cues (contrast 0v3ct). When comparing both groups, we detected an impaired sensitivity in the behavioural response (reaction time and performance improvement) to small rewards (3ct) in the stroke group as well as differences in functional connectivity between the stroke and control group in small reward anticipation (contrasting 0vs.3ct between groups).

To further examine the significance of connectivity changes, we investigated whether there was a link between the altered connectivity difference between connectivity in no-reward and low-reward condition (0vs.3ct) and the altered behavioural performance (RT difference (0vs.3ct)) and learning rate). To this effect, we performed a multivariate regression analysis. The above-mentioned altered cerebral connections (mean coherence; Table 3, Fig. 5) were averaged for each subject. The RT difference and the learning rate were entered in a multivariate regression model together with the parameters age and NIHSS as clinical score of the severity of stroke symptoms (Fig. 1B). This model revealed a highly significant result explaining 50% of measured functional connectivity (R2 = 0.50; adjusted R2 = 0.42, F-value = 5.9; *P* = 0.002). We found that both measures of behavioural performance (RT difference (0vs.3ct)) and learning rate as well as the NIHSS make a significant contribution to the model (Table 4).

**Table 4:** Multiple linear regression of brain connectivity differences of no (0ct) and low (3ct) reward anticipation in stroke patients.

Predictors	B	SE B	β	*P*
Constant	0.15	0.12		0.22
RT 0vs3	0.002	0.0008	0.56	0.009**
Age	−0.002	0.002	−0.22	0.20
NIHSS	−0.02	0.007	−0.46	0.02*
Learning Rate	−0.002	0.0008	−0.45	0.006**

R2 = 0.50; Adjusted R2 = 0.42, F-Value = 5.9; *P* = 0.002.

## 4 Discussion

In this study, we investigated the effects of stroke on the functionality of the reward system, learning ability, and associated brain networks. The results show that stroke-associated changes in the analysed brain regions are related to an altered reward response after stroke. In the following, we will discuss the implications of our findings concerning our understanding of how the brain responds to behavioural, functional, and structural changes after stroke.

### 4.1 The monetary Incentive Delay Task in reward processing analyses

The primary goal of this study was to investigate the acute effects of ischemic lesions on the reward system, the ability to learn and cerebral functional connectivity. The reward response was measured behaviourally as a decrease in reaction time to reward-predicting stimuli (cues) in the well-described reward paradigm Monetary Incentive Delay Task (Knutson et al., 2000) (MID, Fig. 2). The MID acts as a simple reward paradigm in which the reward system is largely unaffected by additional influencing factors (Balodis and Potenza, 2015, DelDonno et al., 2019). Due to its simplicity, the MID is an appropriate analytical tool, especially for older subjects, as no higher cognitive abilities are required (Spaniol et al., 2015). Separate analyses of the cue in MID introduces an appropriate method to study reward processing and to evaluate reward system integrity (Schultz, 2006). The MID represents mechanisms of effort-based decision-making in the context of reaction time responses to varying reward magnitudes (Berridge et al., 2009, Demidenko et al., 2021, Husain and Roiser, 2018). One challenge in studying reward processing is that its different components, such as prediction, receiving, and reinforcement of reward cannot be easily separated. While the MID task represents an effective and robust method for investigating reward system integrity, interpretation of the reaction time data alone may not allow for clear discrimination of these different components. Thus, it may be difficult to determine whether variations in response time reflect a strong emotional response to the reward or simply a decrease in goal directed behaviour.

### 4.2 Reduced reward sensitivity in acute stroke survivors

In acute stroke survivors, we found reduced reward responses to small monetary rewards compared to the control group. A strict correction for multiple comparisons of each single factor of the regression analysis, however would render this effect non-significant which limits the impact of this effect. This is interpreted as decreased reward sensitivity to low reward cues (Fig. 4B, 4E). To our knowledge, this study provides the first evidence for a selectively reduced reward sensitivity to low reward stimuli in the subacute post-stroke phase.

It is worth mentioning that in healthy individuals, the speed of learning is influenced by the magnitude of the reward. Greater reward amounts lead to higher learning processes (Zhang et al., 2018). However, even a small reward stimulus can trigger learning processes in healthy individuals (Zhang et al., 2018). Therefore, the Monetary Incentive Delay (MID) paradigm used in our study could serve as a sensitive tool to identify patients with a diminished response to low reward stimuli and potentially reveal subclinical deficits in reward processing.

Deficits in reward processing in the stroke group may first manifest in response to low reward stimuli finds a potential explanation in the disruption in the interaction of networks involved in reward processing. Even slight disturbances in this network interaction may initially manifest in the response to small reward cues. In contrast, the response to larger reward stimuli, which play a more crucial role, may be better compensated for despite slight network disturbances. he observed group differences in response to low and high reward stimuli in stroke patients may be explained by the presence of evolutionarily stable “surviving- networks such as the reward network (Opitz et al., 2022, Zhang et al., 2014). Similar stability has been observed in fear processing in healthy aging (LaBar et al., 2004).

The changes in functional connectivity during small rewards (0vs3ct) might also reflect acute network dysregulation leading to altered functional connectivity and thus behavioral deficits in reward response. This effect is most notable for DMN networks in healthy aging (Hafkemeijer et al., 2012). Interestingly, similar findings have been observed in patients with Parkinson's disease (PD) who exhibit apathy symptoms. These individuals also show a reduced ability to respond to low reward stimuli, indicating a diminished reward sensitivity. However, it is important to note that these motivational deficits in PD patients cannot be solely attributed to dopaminergic depletion (Le Heron et al., 2018). To evaluate our hypotheses there is further research needed.

Already slight disturbances in this network interaction initially manifest in the response to small reward cues. Meanwhile, the response to larger reward stimuli, which plays an even more crucial role, can be compensated for longer despite slight network disturbances.

Poststroke cognitive impairment, such as a reduced behavioural reward response, is potentially associated with apathy and anhedonia, complicating rehabilitation processes and negatively affecting long-term outcome (Jorge et al., 2010, Mayo et al., 2009). A diminished ability to predict rewards has already been described in chronic stroke status, however, this phenomenon has been demonstrated in acute stroke survivors (Rochat et al., 2013) for the first time in this study, although analogous effects have previously been reported in major depressive disorder (Pizzagalli et al., 2008, Vrieze et al., 2013). Moreover, in line with previous studies, we detected overall slower reaction times in stroke patients (Kulasingham et al., 2021, Rochat et al., 2013, Widmer et al., 2019). According to the reaction time analyses, healthy controls increase their reaction speed with increasing amount of anticipated reward, supporting evidence that the monetary cue in the MID paradigm triggers willingness to exert effort (Broyd et al., 2012, Chiew and Braver, 2011, Dhingra et al., 2020, Oldham et al., 2018, Opitz et al., 2022, Samanez-Larkin et al., 2007, Vaidya et al., 2013).

### 4.3 Reduced reinforcement learning rate after stroke

Since the reward system is a crucial modulator of reinforcement in various cognitive functions, it also plays a critical role in the learning processes (Bowen et al., 2020, Cohen et al., 2016, Schultz et al., 1997, Spaniol et al., 2014). Performance-related reward feedback improves motor skill learning processes and reinforces long-term learning outcome (Boyd et al., 2009, Lam et al., 2013, Vassiliadis et al., 2021, Wachter et al., 2009, Widmer et al., 2022, Widmer et al., 2016). In healthy individuals, the speed of learning depends on the reward magnitude. The greater the amount of reward, the higher the learning processes (Zhang et al., 2018). However, even a small reward stimulus triggers learning processes in healthy people (Zhang et al. 2018).

Especially after stroke, a preserved ability to learn motor skills positively influences rehabilitation success, while deficits in these relearning processes are limiting factors for regaining independence post stroke.

Therefore, we additionally investigated the extent to which reinforcement learning ability is preserved in stroke patients, specifically, whether this ability depended on reward system functionality. In the analysis of the learning rate between the blocks of the MID, severe learning deficits were found in the stroke group compared to the control group (Boyd et al., 2009, Lam et al., 2016) (Fig. 4C, 4F). In previous studies, these deficits were thought to be caused by lesions in the basal ganglia which are known for being involved in motor learning processes (Boyd et al., 2009, Dahms et al., 2020, Schmidt et al., 2008). According to these findings, basal ganglia strokes may lead to disruptions of the connectivity of subcortical networks, impairing circuits between multiple cortico-thalamic regions and the basal ganglia (Boyd et al., 2009, Middleton and Strick, 2000, Middleton and Strick, 2002). In our study sample, minor stroke lesions were distributed in various brain regions, hence comprising a heterogeneous group of patients sharing behaviourally significant deficits in reward sensitivity and reinforcement learning rate (Fig. 4C, 4F). However, the impairments in learning processes detected in this study cannot be explained by the previously described lesion pattern. Learning deficits in stroke patients have recently been shown to be independent from lesion localization and affected hemisphere (Lam et al., 2016, Marsh et al., 2020, Marsh et al., 2022). This is supported by the fact that impairments identified herein go beyond pure motor learning skills. Cognitive deficits following stroke found in the current study are represented by a diminished response to reward predicting cues and are a possible manifestation of reward system dysfunction. Disturbances within this vulnerable system may cause a decreased response to reward or pleasurable stimuli, as well as induce a generally reduced motivational level (Dunlop and Nemeroff, 2007, Vrieze et al., 2013). Due to the strict intertwined connectivity between the reward system and the consolidation of cognitive reinforcement learning processes, learning success may be generally diminished in the event of reward system impairment (Widmer et al., 2019).

### 4.4 Reward prediction network analyses

Comprehension of complex neurocognitive deficits following stroke is scarce. The fact that 50% of stroke survivors suffer from cognitive deficits within the first few months after the event underlines the relevance of understanding the underlying mechanisms for cognitive dysfunction (Edwards et al., 2013, Gorelick and Nyenhuis, 2015, Jacova et al., 2012). Brain network connectivity after stroke has shown a broad spectrum of changes in functional inter-network connectivity patterns accompanied by cognitive deficits (Bournonville et al., 2018, Jaywant and Gunning, 2020, Kulasingham et al., 2021, Lopes et al., 2021, Marsh et al., 2020).

To evaluate changes in the behavioural reward response at the neural level, this study additionally conducted connectivity analyses using high temporal resolution in MEG measurements during reward anticipation.

Connectivity changes detected after stroke have previously been shown in global brain network alterations as well as in changes of specific subnetworks (Jaywant et al., 2022, Li et al., 2021, Siegel et al., 2016, Sun et al., 2018, Zhu et al., 2017). Those global network changes have been discussed as underlying mechanisms for cognitive impairment and executive dysfunction following stroke (Jaywant et al., 2022, Marsh et al., 2020).

Our study design allows a direct detection of cortical responses with high temporal resolution to reward prediction. Herein, the integrity of the reward network could be assessed in an isolated manner by fading out baseline activity and confounding factors. Modern connectivity analyses allow evaluation of cortical brain network functions. To examine the neural communication processes, we used coherence as a well-established method. The high temporal resolution of MEG measurements can be used to analyse brain connectivity at the time of reward anticipation (Bastos and Schoffelen, 2015, Fries, 2015, Srinivasan et al., 2007). In our MEG functional connectivity analyses, we identified significant group differences in reward anticipation between the left frontal region and the left temporoparietal region, as well as between the right frontal region and the right temporoparietal region in the theta bands (Fig. 5). Frontal theta band oscillations are a relevant marker for monitoring cognitive control, attentional networks and decision making (Cohen and Donner, 2013, Duprez et al., 2020, Lin et al., 2022). Impairment of reinforcement learning and a diminished reward sensitivity have been hypothesized to manifest in alterations in midfrontal theta band oscillations (Azanova et al., 2021, Cohen and Cavanagh, 2011, Lin et al., 2022).

A reduced reward sensitivity representing the main stroke-related alteration is associated with group differences in the functional connectivity (coherence) between the left frontal region and the right temporoparietal region (TPR), the right frontal region (FR) and the left temporoparietal region (TPL) and the FR and the TPR for small reward cues (Table 3, Fig. 5). Our regression model used herein is able to answer the essential question of whether there is a relationship between connectivity alterations and behavioural changes.

Previous studies detected connectivity alterations in frontal and frontoparietal brain areas in stroke patients (Grefkes et al., 2008, Kulasingham et al., 2021, Westlake and Nagarajan, 2011, Zhang et al., 2017, Zhu et al., 2014, Zhu et al., 2017). The medial orbitofrontal cortex in particular has been described extensively as an anatomical hub of the reward system (Berridge and Kringelbach, 2015, Haber and Knutson, 2010). Prefrontal and parietal areas are part of the cognitive (CCN) and default mode network (DMN), and temporal brain areas are additionally described as part of the DMN (Greicius et al., 2003, Jaywant et al., 2022). These areas broadly correspond to MEG-regions in this study and translated as FR/FL and TPL/TPR regions (Fig. 3).

Alterations in brain networks (e.g., CCN, DMN) as well as functional changes in the reward network even after minor strokes could be a possible explanation for cognitive impairments following stroke (Jaywant and Gunning, 2020, Marsh et al., 2020). The main functions of the CCN include coordination of goal-directed behaviour, focusing attention and fading out confounding factors (Cole and Schneider, 2007, Jaywant et al., 2022, Niendam et al., 2012). In healthy individuals, cortical areas of the DMN are frequently deactivated during active tasks and activated in attentional tasks (Raichle et al., 2001). Indeed, altered functional connectivity of the cognitive control and default mode network after stroke (Egorova et al., 2018, Tuladhar et al., 2013, Zhang et al., 2017) can be inferred from our MEG data (Fig. 5).

### 4.5 The relationship between learning disabilities and diminished rewards ability

Compared between groups, stroke patients showed an impaired sensitivity in the behavioural reward performance, a reduced learning rate, and altered functional connectivity, mainly due to small reward anticipation (contrasting 0vs.3ct between groups). Multivariate regression analysis showed that the changes in connectivity between the no-reward (0ct) and low-reward (3ct) conditions were associated with the differences in reaction time and learning rate (Table 4). That means that all three effects (reduced reward sensitivity, reduced learning ability, and altered cerebral connectivity) were found not only to be different in stroke patients compared to healthy subjects but also that the disturbance's strength is tightly connected between these parameters. This connection suggests that the structural lesion due to the acute stroke induces reward network dysfunction, leading to impaired behavioral reward functioning and an altered ability for motor learning. These findings are representative of a general pattern in mild strokes and are independent of the specific lesion localization. Our study model revealed that a higher impairment in connectivity and behavioral reward response significantly correlates with higher NIHSS scores, indicating a potential link between cognitive and motor deficits following stroke. These findings suggest that a more comprehensive examination of the interplay between various aspects of stroke pathophysiology and reward system function could improve diagnosis and therapeutic development. These results represent an important entry point for stroke rehabilitation to identify the reduced learning capacity after stroke and implement individualized rehabilitation exercises.

### 4.6 Methodical limitations

Reward experiences in the form of secondary reward stimuli are always dependent on the individual level of valuation, but monetary rewards provided herein, allow a good gradation of the reward magnitude between high and low rewards compared exclusively to positive feedback. Due to the high number of trials (300 complete tasks, in three equal blocks with 100 trials each) the structure of our MID may have led to a monotonous task experience, which can negatively affect subjects' attention (Bjork et al., 2010). Participants, especially in the stroke group, reported a reduced attentional level in the third block. The effect of visual attention as another influencing factor on reward response was reduced by temporally adjusting the displayed reward cue within the paradigm (Cheng et al., 2021).

We tested larger areas due to the limited level of spatial resolution in MEG imaging measurement and varying interindividual network expressions (Samuelsson et al., 2021). Coherence connectivity analyses only provide limited robustness due to heterogeneous data quality and the low number of participating subjects (Bastos and Schoffelen, 2015, Fries, 2015). Results for significance should therefore be interpreted with caution. In contrast, the behavioural analyses of reaction times and learning rates can be interpreted with greater reliability. The large number of values used in the GEE model (*n* = 10747) reduced the individual statistical bias of each subject. Nevertheless, the significant interaction effect between the low reward cue (3ct) and the subject group (stroke vs. healthy controls) (m = 8.61, stderr = 3.72, *P* = 0.021) is not highly significant and would therefore not survive corrections for multiple comparisons.

Since all patients performed the task with the unaffected hand regardless of the presence of hemiparesis, the changes detected in the responses in the stroke group cannot be explained by motor limitations.

## 5 Conclusion

In conclusion, our results demonstrate that patients in the acute phase following stroke show reduced reward sensitivity, reduced ability to learn and an altered cerebral connectivity pattern. All three effects are tightly coupled and deviate strongly from the control group with respect to low reward conditions. These findings are representative for a general pattern of minor stroke and are independent from specific lesion localization in classic anatomic reward-related brain regions. The question of whether these brain network changes appear transient or chronical remains open. Therefore, long term studies are required to assess the network outcome post stroke (Nicolas et al., 2021). For stroke rehabilitation, these results set a key point to identify reduced learning capacity after stroke and accordingly to individually customize recovery exercises.

## CRediT authorship contribution statement

**Franziska Wagner:** Conceptualization, Data curation, Formal analysis, Funding acquisition, Methodology, Project administration, Supervision, Writing – original draft, Writing – review & editing. **Jenny Rogenz:** Data curation, Formal analysis, Funding acquisition, Visualization, Writing – original draft, Writing – review & editing. **Laura Opitz:** Data curation, Formal analysis. **Johanna Maas:** Data curation, Formal analysis. **Alexander Schmidt:** Data curation, Formal analysis, Validation, Visualization, Writing – review & editing. **Stefan Brodoehl:** Formal analysis, Methodology, Writing – review & editing. **Markus Ullsperger:** Writing – review & editing. **Carsten M. Klingner:** Conceptualization, Data curation, Formal analysis, Project administration, Supervision, Validation, Writing – review & editing.

## Declaration of Competing Interest

The authors declare that they have no known competing financial interests or personal relationships that could have appeared to influence the work reported in this paper.

## Figures and Tables

**Supplementary Table 1:**
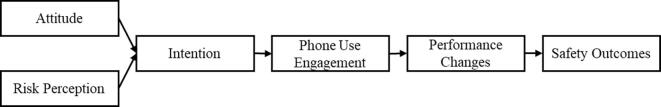
Overview of candidate GEE models. M1 and M2 correspond to the full saturated models, including the three-level factor cue, three-level factor block, and 2-level factor group, with all interaction terms considered but employing different correlation structure specifications. Based on the Quasi-Likelihood Information Criterion (QIC) and Corrected Quasi-Likelihood Information Criterion (CIC) values, which are utilized to determine an appropriate correlation structure, M2 demonstrates a better fit to the data, rendering M1 no longer relevant. M3 represents the model without the three-way interaction, additionally removing the interaction term between cue and block, and performs comparably to M1. Subsequent candidate models involve extensions or combinations with the confounder variables age, BDI, MoCA, and gender. M8 and M9 exhibit the lowest QICu values, indicating their suitability as the most appropriate candidate models. Nevertheless, based on an analysis of variance (ANOVA) comparing these two models, it is evident that M9 does not yield a significant improvement in model performance. Consequently, M8 was selected as the preferred model for further analyses; Model parameter estimates are presented in the top section, model selection criteria in the middle section, and model information in the bottom section); Statistical significance is denoted as follows:*** p < 0.001; ** p < 0.01; * p < 0.05.

## Data Availability

Data will be made available on request.

## References

[b0005] Atteih S., Mellon L., Hall P., Brewer L., Horgan F., Williams D., Hickey A., group, A.-S.s. (2015). Implications of stroke for caregiver outcomes: findings from the ASPIRE-S study. Int. J. Stroke.

[b0010] Ayerbe L., Ayis S., Wolfe C.D., Rudd A.G. (2013). Natural history, predictors and outcomes of depression after stroke: systematic review and meta-analysis. Br. J. Psychiatry.

[b0015] Azanova M., Herrojo Ruiz M., Belianin A.V., Klucharev V., Nikulin V.V. (2021). Resting-state theta oscillations and reward sensitivity in risk taking. Front. Neurosci..

[b0020] Balodis I.M., Potenza M.N. (2015). Anticipatory reward processing in addicted populations: a focus on the monetary incentive delay task. Biol. Psychiatry.

[b0025] Bastos A.M., Schoffelen J.M. (2015). A tutorial review of functional connectivity analysis methods and their interpretational pitfalls. Front. Syst. Neurosci..

[b0030] Beck A.T., Steer R.A., Brown G.K. (1996).

[b0035] Benjamin E.J., Muntner P., Alonso A., Bittencourt M.S., Callaway C.W., Carson A.P., Chamberlain A.M., Chang A.R., Cheng S., Das S.R., Delling F.N., Djousse L., Elkind M.S.V., Ferguson J.F., Fornage M., Jordan L.C., Khan S.S., Kissela B.M., Knutson K.L., Kwan T.W., Lackland D.T., Lewis T.T., Lichtman J.H., Longenecker C.T., Loop M.S., Lutsey P.L., Martin S.S., Matsushita K., Moran A.E., Mussolino M.E., O'Flaherty M., Pandey A., Perak A.M., Rosamond W.D., Roth G.A., Sampson U.K.A., Satou G.M., Schroeder E.B., Shah S.H., Spartano N.L., Stokes A., Tirschwell D.L., Tsao C.W., Turakhia M.P., VanWagner L.B., Wilkins J.T., Wong S.S., Virani S.S. (2019). Heart Disease and Stroke statistics-2019 update: a report From the American Heart Association. Circulation.

[b0040] Berridge K.C., Kringelbach M.L. (2015). Pleasure systems in the brain. Neuron.

[b0045] Berridge K.C., Robinson T.E., Aldridge J.W. (2009). Dissecting components of reward: 'liking', 'wanting', and learning. Curr. Opin. Pharmacol..

[b0050] Bjork J.M., Smith A.R., Chen G., Hommer D.W. (2010). Adolescents, adults and rewards: comparing motivational neurocircuitry recruitment using fMRI. PLoS One.

[b0055] Bonkhoff, A.K., Schirmer, M.D., Bretzner, M., Etherton, M., Donahue, K., Tuozzo, C., Nardin, M., Giese, A.-K., Wu, O., D. Calhoun, V., Grefkes, C., Rost, N.S., 2021. Abnormal dynamic functional connectivity is linked to recovery after acute ischemic stroke. Hum. Brain Mapp. 42, 2278-2291. doi: 10.1002/hbm.25366. 10.1002/hbm.25366 PMC8046120 33650754

[b0060] Botvinick M., Braver T. (2015). Motivation and cognitive control: from behavior to neural mechanism. Annu. Rev. Psychol..

[b0065] Bour A., Rasquin S., Limburg M., Verhey F. (2011). Depressive symptoms and executive functioning in stroke patients: a follow-up study. Int. J. Geriatr. Psychiatry.

[b0070] Bournonville C., Henon H., Dondaine T., Delmaire C., Bombois S., Mendyk A.M., Cordonnier C., Moulin S., Leclerc X., Bordet R., Lopes R. (2018). Identification of a specific functional network altered in poststroke cognitive impairment. Neurology.

[b0075] Bowen H.J., Gallant S.N., Moon D.H. (2020). Influence of reward motivation on directed forgetting in younger and older adults. Front. Psychol..

[b0080] Bowyer S.M. (2016). Coherence a measure of the brain networks: past and present. Neuropsychiatric Electrophysiology.

[b0085] Boyd L.A., Edwards J.D., Siengsukon C.S., Vidoni E.D., Wessel B.D., Linsdell M.A. (2009). Motor sequence chunking is impaired by basal ganglia stroke. Neurobiol. Learn. Mem..

[b0090] Brott T., Adams H.P., Olinger C.P., Marler J.R., Barsan W.G., Biller J., Spilker J., Holleran R., Eberle R., Hertzberg V. (1989). Measurements of acute cerebral infarction: a clinical examination scale. Stroke.

[b0095] Broyd S.J., Richards H.J., Helps S.K., Chronaki G., Bamford S., Sonuga-Barke E.J. (2012). An electrophysiological monetary incentive delay (e-MID) task: a way to decompose the different components of neural response to positive and negative monetary reinforcement. J. Neurosci. Methods.

[b0100] Carson A.J., MacHale S., Allen K., Lawrie S.M., Dennis M., House A., Sharpe M. (2000). Depression after stroke and lesion location: a systematic review. Lancet.

[b0105] Chau B.K.H., Jarvis H., Law C.K., Chong T.T. (2018). Dopamine and reward: a view from the prefrontal cortex. Behav. Pharmacol..

[b0110] Cheng P.X., Rich A.N., Le Pelley M.E. (2021). Reward rapidly enhances visual perception. Psychol. Sci..

[b0115] Chiew K.S., Braver T.S. (2011). Positive affect versus reward: emotional and motivational influences on cognitive control. Front. Psychol..

[b0120] Cohen M.X. (2011). Error-related medial frontal theta activity predicts cingulate-related structural connectivity. Neuroimage.

[b0125] Cohen M.X., Cavanagh J.F. (2011). Single-trial regression elucidates the role of prefrontal theta oscillations in response conflict. Front. Psychol..

[b0130] Cohen M.X., Donner T.H. (2013). Midfrontal conflict-related theta-band power reflects neural oscillations that predict behavior. J. Neurophysiol..

[b0135] Cohen M.S., Rissman J., Suthana N.A., Castel A.D., Knowlton B.J. (2016). Effects of aging on value-directed modulation of semantic network activity during verbal learning. Neuroimage.

[b0140] Cole M.W., Schneider W. (2007). The cognitive control network: Integrated cortical regions with dissociable functions. Neuroimage.

[b0145] Cooley J.W., Tukey J.W. (1965). An algorithm for the machine calculation of complex Fourier series. Mathematics of Computation.

[b0150] Corbetta M., Shulman G.L. (2002). Control of goal-directed and stimulus-driven attention in the brain. Nat. Rev. Neurosci..

[b0155] Dahms C., Brodoehl S., Witte O.W., Klingner C.M. (2020). The importance of different learning stages for motor sequence learning after stroke. Hum. Brain Mapp..

[b0160] DelDonno S.R., Karstens A.J., Cerny B., Kling L.R., Jenkins L.M., Stange J.P., Nusslock R., Shankman S.A., Langenecker S.A. (2019). The Titrated Monetary Incentive Delay Task: Sensitivity, convergent and divergent validity, and neural correlates in an RDoC sample. J. Clin. Exp. Neuropsychol..

[b0165] Demidenko M.I., Weigard A.S., Ganesan K., Jang H., Jahn A., Huntley E.D., Keating D.P. (2021). Interactions between methodological and interindividual variability: How Monetary Incentive Delay (MID) task contrast maps vary and impact associations with behavior. Brain Behav..

[b0170] Dhingra I., Zhang S., Zhornitsky S., Le T.M., Wang W., Chao H.H., Levy I., Li C.R. (2020). The effects of age on reward magnitude processing in the monetary incentive delay task. Neuroimage.

[b0175] Dunlop B.W., Nemeroff C.B. (2007). The role of dopamine in the pathophysiology of depression. Arch. Gen. Psychiatry.

[b0180] Duprez J., Gulbinaite R., Cohen M.X. (2020). Midfrontal theta phase coordinates behaviorally relevant brain computations during cognitive control. Neuroimage.

[b0185] Edwards J.D., Jacova C., Sepehry A.A., Pratt B., Benavente O.R. (2013). A quantitative systematic review of domain-specific cognitive impairment in lacunar stroke. Neurology.

[b0190] Egorova N., Cumming T., Shirbin C., Veldsman M., Werden E., Brodtmann A. (2018). Lower cognitive control network connectivity in stroke participants with depressive features. Transl Psychiatry.

[b0195] Engel A.K., Fries P. (2010). Beta-band oscillations–signalling the status quo?. Curr. Opin. Neurobiol..

[b0200] EuroQol G. (1990). EuroQol–a new facility for the measurement of health-related quality of life. Health Policy.

[b0205] Fellows L.K. (2004). The cognitive neuroscience of human decision making: a review and conceptual framework. Behav. Cogn. Neurosci. Rev..

[b0210] Fries P. (2015). Rhythms for cognition: communication through coherence. Neuron.

[b0215] Friston K.J. (2011). Functional and effective connectivity: a review. Brain Connect..

[b0220] Fugl-Meyer A.R., Jaasko L., Leyman I., Olsson S., Steglind S. (1975). The post-stroke hemiplegic patient. 1. a method for evaluation of physical performance. Scand. J. Rehabil. Med..

[b0225] Gerlach K.D., Spreng R.N., Madore K.P., Schacter D.L. (2014). Future planning: default network activity couples with frontoparietal control network and reward-processing regions during process and outcome simulations. Soc. Cogn. Affect. Neurosci..

[b0230] Ghose S.S., Williams L.S., Swindle R.W. (2005). Depression and other mental health diagnoses after stroke increase inpatient and outpatient medical utilization three years poststroke. Med. Care.

[b0235] Gorelick P.B., Nyenhuis D. (2015). Stroke and cognitive decline. JAMA.

[b0240] Grefkes C., Nowak D.A., Eickhoff S.B., Dafotakis M., Kust J., Karbe H., Fink G.R. (2008). Cortical connectivity after subcortical stroke assessed with functional magnetic resonance imaging. Ann. Neurol..

[b0245] Greicius M.D., Krasnow B., Reiss A.L., Menon V. (2003). Functional connectivity in the resting brain: a network analysis of the default mode hypothesis. Proc. Natl. Acad. Sci. U. S. A..

[b0250] Haber S.N. (2016). Corticostriatal circuitry. Dialogues Clin. Neurosci..

[b0255] Haber S.N., Knutson B. (2010). The reward circuit: linking primate anatomy and human imaging. Neuropsychopharmacology.

[b0260] Hafkemeijer A., van der Grond J., Rombouts S.A.R.B. (2012). Imaging the default mode network in aging and dementia. Biochim. Biophys. Acta (BBA) – Mol. Basis Disease.

[b0265] Halekoh U., Højsgaard S., Yan J. (2006). TheRPackagegeepackfor generalized estimating equations. J. Stat. Softw..

[b0270] Husain M., Roiser J.P. (2018). Neuroscience of apathy and anhedonia: a transdiagnostic approach. Nat. Rev. Neurosci..

[b0275] Ikemoto S. (2010). Brain reward circuitry beyond the mesolimbic dopamine system: a neurobiological theory. Neurosci. Biobehav. Rev..

[b0280] Jacova C., Pearce L.A., Costello R., McClure L.A., Holliday S.L., Hart R.G., Benavente O.R. (2012). Cognitive impairment in lacunar strokes: the SPS3 trial. Ann. Neurol..

[b0285] Jaywant A., DelPonte L., Kanellopoulos D., O'Dell M.W., Gunning F.M. (2022). The structural and functional neuroanatomy of post-stroke depression and executive dysfunction: a review of neuroimaging findings and implications for treatment. J. Geriatr. Psychiatry Neurol..

[b0290] Jaywant A., Gunning F.M., Lazar R.M., Pavol M.A., Browndyke J.N. (2020). Neurovascular Neuropsychology.

[b0295] Jorge R.E., Starkstein S.E., Robinson R.G. (2010). Apathy following stroke. Can. J. Psychiatry.

[b0300] Kasner S.E. (2006). Clinical interpretation and use of stroke scales. Lancet Neurol..

[b0305] Kelly-Hayes M., Beiser A., Kase C.S., Scaramucci A., D’Agostino R.B., Wolf P.A. (2003). The influence of gender and age on disability following ischemic stroke: the Framingham study. J. Stroke Cerebrovasc. Dis..

[b0310] Kida T., Tanaka E., Kakigi R. (2015). Multi-dimensional dynamics of human electromagnetic brain activity. Front. Hum. Neurosci..

[b0315] Kitago T., Krakauer J.W. (2013). Motor learning principles for neurorehabilitation. Handb. Clin. Neurol..

[b0320] Knutson B., Westdorp A., Kaiser E., Hommer D. (2000). FMRI visualization of brain activity during a monetary incentive delay task. Neuroimage.

[b0325] Krakauer J.W. (2006). Motor learning: its relevance to stroke recovery and neurorehabilitation. Curr. Opin. Neurol..

[b0330] Kulasingham, J.P., Brodbeck, C., Khan, S., Marsh, E.B., Simon, J.Z., 2021. Bilaterally Reduced Rolandic Beta Band Activity in Minor Stroke Patients. bioRxiv, 2021.2010.2015.464457. doi: 10.1101/2021.10.15.464457. 10.3389/fneur.2022.819603 PMC8996122 35418932

[b0335] Kutlubaev M.A., Hackett M.L. (2014). Part II: predictors of depression after stroke and impact of depression on stroke outcome: an updated systematic review of observational studies. Int. J. Stroke.

[b0340] LaBar K.S., Cook C.A., Torpey D.C., Welsh-Bohmer K.A. (2004). Impact of healthy aging on awareness and fear conditioning. Behav. Neurosci..

[b0345] Lam J.M., Wachter T., Globas C., Karnath H.O., Luft A.R. (2013). Predictive value and reward in implicit classification learning. Hum. Brain Mapp..

[b0350] Lam J.M., Globas C., Hosp J.A., Karnath H.O., Wachter T., Luft A.R. (2016). Impaired implicit learning and feedback processing after stroke. Neuroscience.

[b0355] Lammel S., Hetzel A., Häckel O., Jones I., Liss B., Roeper J. (2008). Unique properties of mesoprefrontal neurons within a dual mesocorticolimbic dopamine system. Neuron.

[b0360] Le Heron C., Plant O., Manohar S., Ang Y.-S., Jackson M., Lennox G., Hu M.T., Husain M. (2018). Distinct effects of apathy and dopamine on effort-based decision-making in Parkinson’s disease. Brain.

[b0365] Li Y., Yu Z., Wu P., Chen J. (2021). The disrupted topological properties of structural networks showed recovery in ischemic stroke patients: a longitudinal design study. BMC Neurosci..

[b0370] Liang K.-Y., Zeger S.L. (1986). Longitudinal data analysis using generalized linear models. Biometrika.

[b0375] Lin M.H., Liran O., Bauer N., Baker T.E. (2022). Scalp recorded theta activity is modulated by reward, direction, and speed during virtual navigation in freely moving humans. Sci. Rep..

[b0380] Lopes R., Bournonville C., Kuchcinski G., Dondaine T., Mendyk A.M., Viard R., Pruvo J.P., Henon H., Georgakis M.K., Duering M., Dichgans M., Cordonnier C., Leclerc X., Bordet R. (2021). Prediction of long-term cognitive functions after minor stroke, using functional connectivity. Neurology.

[b0385] Luft C.D., Nolte G., Bhattacharya J. (2013). High-learners present larger mid-frontal theta power and connectivity in response to incorrect performance feedback. J. Neurosci..

[b0390] Marsh E.B., Brodbeck C., Llinas R.H., Mallick D., Kulasingham J.P., Simon J.Z., Llinas R.R. (2020). Poststroke acute dysexecutive syndrome, a disorder resulting from minor stroke due to disruption of network dynamics. Proc. Natl. Acad. Sci. U. S. A..

[b0395] Marsh E.B., Khan S., Llinas R.H., Walker K.A., Brandt J. (2022). Multidomain cognitive dysfunction after minor stroke suggests generalized disruption of cognitive networks. Brain Behav.

[b0400] Mayo N.E., Fellows L.K., Scott S.C., Cameron J., Wood-Dauphinee S. (2009). A longitudinal view of apathy and its impact after stroke. Stroke.

[b0405] Meyer B.C., Hemmen T.M., Jackson C.M., Lyden P.D. (2002). Modified National Institutes of Health Stroke Scale for use in stroke clinical trials: prospective reliability and validity. Stroke.

[b0410] Middleton F.A., Strick P.L. (2000). Basal ganglia and cerebellar loops: motor and cognitive circuits. Brain Res. Brain Res. Rev..

[b0415] Middleton F.A., Strick P.L. (2002). Basal-ganglia 'projections' to the prefrontal cortex of the primate. Cereb. Cortex.

[b0420] Narushima K., Kosier J.T., Robinson R.G. (2003). A reappraisal of poststroke depression, intra- and inter-hemispheric lesion location using meta-analysis. J. Neuropsychiatry Clin. Neurosci..

[b0425] Nasreddine Z.S., Phillips N.A., Bedirian V., Charbonneau S., Whitehead V., Collin I., Cummings J.L., Chertkow H. (2005). The Montreal Cognitive Assessment, MoCA: a brief screening tool for mild cognitive impairment. J. Am. Geriatr. Soc..

[b0430] Nicolas K., Goodin P., Visser M.M., Michie P.T., Bivard A., Levi C., Parsons M.W., Karayanidis F. (2021). Altered functional connectivity and cognition persists 4 years after a transient ischemic attack or minor stroke. Front. Neurol..

[b0435] Niendam T.A., Laird A.R., Ray K.L., Dean Y.M., Glahn D.C., Carter C.S. (2012). Meta-analytic evidence for a superordinate cognitive control network subserving diverse executive functions. Cogn. Affect. Behav. Neurosci..

[b0440] O'Doherty J.P., Cockburn J., Pauli W.M. (2017). Learning, reward, and decision making. Annu. Rev. Psychol..

[b0445] Oldfield R.C. (1971). The assessment and analysis of handedness: the Edinburgh inventory. Neuropsychologia.

[b0450] Oldham S., Murawski C., Fornito A., Youssef G., Yucel M., Lorenzetti V. (2018). The anticipation and outcome phases of reward and loss processing: A neuroimaging meta-analysis of the monetary incentive delay task. Hum. Brain Mapp..

[b0455] Oostenveld R., Fries P., Maris E., Schoffelen J.M. (2011). FieldTrip: Open source software for advanced analysis of MEG, EEG, and invasive electrophysiological data. Comput. Intell. Neurosci..

[b0460] Opitz L., Wagner F., Rogenz J., Maas J., Schmidt A., Brodoehl S., Klingner C.M. (2022). Still wanting to win: reward system stability in healthy aging. Front. Aging Neurosci..

[b0465] Pan C., Li G., Sun W., Miao J., Qiu X., Lan Y., Wang Y., Wang H., Zhu Z., Zhu S. (2022). Neural substrates of poststroke depression: current opinions and methodology trends. Front. Neurosci..

[b0470] Parro C., Dixon M.L., Christoff K. (2018). The neural basis of motivational influences on cognitive control. Hum. Brain Mapp..

[b0475] Pereda E., Quiroga R.Q., Bhattacharya J. (2005). Nonlinear multivariate analysis of neurophysiological signals. Prog. Neurobiol..

[b0480] Pessoa L., Engelmann J.B. (2010). Embedding reward signals into perception and cognition. Front. Neurosci..

[b0485] Pizzagalli D.A., Iosifescu D., Hallett L.A., Ratner K.G., Fava M. (2008). Reduced hedonic capacity in major depressive disorder: evidence from a probabilistic reward task. J. Psychiatr. Res..

[b0490] Quattrocchi G., Greenwood R., Rothwell J.C., Galea J.M., Bestmann S. (2017). Reward and punishment enhance motor adaptation in stroke. J. Neurol. Neurosurg. Psychiatry.

[b0495] Raichle M.E., MacLeod A.M., Snyder A.Z., Powers W.J., Gusnard D.A., Shulman G.L. (2001). A default mode of brain function. Proc. Natl. Acad. Sci. U. S. A..

[b0500] Rankin J. (1957). Cerebral vascular accidents in patients over the age of 60. II. Prognosis. Scott. Med. J..

[b0505] Rochat L., Van der Linden M., Renaud O., Epiney J.B., Michel P., Sztajzel R., Spierer L., Annoni J.M. (2013). Poor reward sensitivity and apathy after stroke: implication of basal ganglia. Neurology.

[b0510] Samanez-Larkin G.R., Gibbs S.E., Khanna K., Nielsen L., Carstensen L.L., Knutson B. (2007). Anticipation of monetary gain but not loss in healthy older adults. Nat. Neurosci..

[b0515] Samuelsson J.G., Peled N., Mamashli F., Ahveninen J., Hamalainen M.S. (2021). Spatial fidelity of MEG/EEG source estimates: A general evaluation approach. Neuroimage.

[b0520] Schmidt L., d'Arc B.F., Lafargue G., Galanaud D., Czernecki V., Grabli D., Schupbach M., Hartmann A., Levy R., Dubois B., Pessiglione M. (2008). Disconnecting force from money: effects of basal ganglia damage on incentive motivation. Brain.

[b0525] Schultz W. (2000). Multiple reward signals in the brain. Nat. Rev. Neurosci..

[b0530] Schultz W. (2006). Behavioral theories and the neurophysiology of reward. Annu. Rev. Psychol..

[b0535] Schultz W. (2015). Neuronal reward and decision signals: from theories to data. Physiol. Rev..

[b0540] Schultz W., Dayan P., Montague P.R. (1997). A neural substrate of prediction and reward. Science.

[b0545] Shi Y., Yang D., Zeng Y., Wu W. (2017). Risk factors for post-stroke depression: a meta-analysis. Front. Aging Neurosci..

[b0550] Siegel J.S., Ramsey L.E., Snyder A.Z., Metcalf N.V., Chacko R.V., Weinberger K., Baldassarre A., Hacker C.D., Shulman G.L., Corbetta M. (2016). Disruptions of network connectivity predict impairment in multiple behavioral domains after stroke. Proc. Natl. Acad. Sci. U. S. A..

[b0555] Spaniol J., Schain C., Bowen H.J. (2014). Reward-enhanced memory in younger and older adults. J. Gerontol. B Psychol. Sci. Soc. Sci..

[b0560] Spaniol J., Bowen H.J., Wegier P., Grady C. (2015). Neural responses to monetary incentives in younger and older adults. Brain Res..

[b0565] Srinivasan R., Winter W.R., Ding J., Nunez P.L. (2007). EEG and MEG coherence: measures of functional connectivity at distinct spatial scales of neocortical dynamics. J. Neurosci. Methods.

[b0570] Subramanian S.K., Massie C.L., Malcolm M.P., Levin M.F. (2010). Does provision of extrinsic feedback result in improved motor learning in the upper limb poststroke? A systematic review of the evidence. Neurorehabil. Neural Repair.

[b0575] Sun C., Yang F., Wang C., Wang Z., Zhang Y., Ming D., Du J. (2018). Mutual information-based brain network analysis in post-stroke patients with different levels of depression. Front. Hum. Neurosci..

[b0580] Taulu S., Simola J., Kajola M. (2005). Applications of the signal space separation method. IEEE Trans. Signal Process..

[b0585] Tay J., Lisiecka-Ford D.M., Hollocks M.J., Tuladhar A.M., Barrick T.R., Forster A., O’Sullivan M.J., Husain M., de Leeuw F.-E., Morris R.G., Markus H.S. (2020). Network neuroscience of apathy in cerebrovascular disease. Prog. Neurobiol..

[b0590] Tay J., Morris R.G., Markus H.S. (2021). Apathy after stroke: Diagnosis, mechanisms, consequences, and treatment. Int. J. Stroke.

[b0595] Tewarie P., Liuzzi L., O'Neill G.C., Quinn A.J., Griffa A., Woolrich M.W., Stam C.J., Hillebrand A., Brookes M.J. (2019). Tracking dynamic brain networks using high temporal resolution MEG measures of functional connectivity. Neuroimage.

[b0600] Tuladhar A.M., Snaphaan L., Shumskaya E., Rijpkema M., Fernandez G., Norris D.G., de Leeuw F.E. (2013). Default mode network connectivity in stroke patients. PLoS One.

[b0605] Ullsperger M., Fischer A.G., Nigbur R., Endrass T. (2014). Neural mechanisms and temporal dynamics of performance monitoring. Trends Cogn. Sci..

[b0610] Vaidya J.G., Knutson B., O'Leary D.S., Block R.I., Magnotta V. (2013). Neural sensitivity to absolute and relative anticipated reward in adolescents. PLoS One.

[b0615] van Vliet P.M., Wulf G. (2006). Extrinsic feedback for motor learning after stroke: what is the evidence?. Disabil. Rehabil..

[b0620] Vassiliadis P., Derosiere G., Dubuc C., Lete A., Crevecoeur F., Hummel F.C., Duque J. (2021). Reward boosts reinforcement-based motor learning. iScience.

[b0625] Vincent J.L., Kahn I., Snyder A.Z., Raichle M.E., Buckner R.L. (2008). Evidence for a frontoparietal control system revealed by intrinsic functional connectivity. J. Neurophysiol..

[b0630] Virani, S.S., Alonso, A., Benjamin, E.J., Bittencourt, M.S., Callaway, C.W., Carson, A.P., Chamberlain, A.M., Chang, A.R., Cheng, S., Delling, F.N., Djousse, L., Elkind, M.S.V., Ferguson, J.F., Fornage, M., Khan, S.S., Kissela, B.M., Knutson, K.L., Kwan, T.W., Lackland, D.T., Lewis, T.T., Lichtman, J.H., Longenecker, C.T., Loop, M.S., Lutsey, P.L., Martin, S.S., Matsushita, K., Moran, A.E., Mussolino, M.E., Perak, A.M., Rosamond, W.D., Roth, G.A., Sampson, U.K.A., Satou, G.M., Schroeder, E.B., Shah, S.H., Shay, C.M., Spartano, N.L., Stokes, A., Tirschwell, D.L., VanWagner, L.B., Tsao, C.W., American Heart Association Council on, E., Prevention Statistics, C., Stroke Statistics, S., 2020. Heart Disease and Stroke Statistics-2020 Update: A Report From the American Heart Association. Circulation 141, e139-e596. doi: 10.1161/CIR.0000000000000757. 10.1161/CIR.0000000000000757 31992061

[b0635] Vrieze E., Pizzagalli D.A., Demyttenaere K., Hompes T., Sienaert P., de Boer P., Schmidt M., Claes S. (2013). Reduced reward learning predicts outcome in major depressive disorder. Biol. Psychiatry.

[b0640] Wachter T., Lungu O.V., Liu T., Willingham D.T., Ashe J. (2009). Differential effect of reward and punishment on procedural learning. J. Neurosci..

[b0645] Ware Jr, J.E., 1999. SF-36 Health Survey. The use of psychological testing for treatment planning and outcomes assessment, 2nd ed. Lawrence Erlbaum Associates Publishers, Mahwah, NJ, US, pp. 1227-1246.

[b0650] Westlake K.P., Nagarajan S.S. (2011). Functional connectivity in relation to motor performance and recovery after stroke. Front. Syst. Neurosci..

[b0655] Widmer, M., Ziegler, N., Held, J., Luft, A., Lutz, K., 2016. Chapter 13 – Rewarding feedback promotes motor skill consolidation via striatal activity. In: Studer, B., Knecht, S. (Eds.), Prog. Brain Res. Elsevier, pp. 303-323. 10.1016/bs.pbr.2016.05.006 27926445

[b0660] Widmer M., Lutz K., Luft A.R. (2019). Reduced striatal activation in response to rewarding motor performance feedback after stroke. Neuroimage Clin..

[b0665] Widmer M., Held J.P.O., Wittmann F., Valladares B., Lambercy O., Sturzenegger C., Palla A., Lutz K., Luft A.R. (2022). Reward during arm training improves impairment and activity after stroke: a randomized controlled trial. Neurorehabil. Neural Repair.

[b0670] Wise R.A. (2002). Brain reward circuitry: insights from unsensed incentives. Neuron.

[b0675] Zhang P., Xu Q., Dai J., Wang J., Zhang N., Luo Y. (2014). Dysfunction of affective network in post ischemic stroke depression: a resting-state functional magnetic resonance imaging study. Biomed. Res. Int..

[b0680] Zhang P., Hou F., Yan F.F., Xi J., Lin B.R., Zhao J., Yang J., Chen G., Zhang M.Y., He Q., Dosher B.A., Lu Z.L., Huang C.B. (2018). High reward enhances perceptual learning. J. Vis..

[b0685] Zhang Y., Wang L., Yang J., Yan R., Zhang J., Sang L., Li P., Liu H., Qiu M. (2017). Abnormal functional networks in resting-state of the sub-cortical chronic stroke patients with hemiplegia. Brain Res..

[b0690] Zhu Y., Bai L., Liang P., Kang S., Gao H., Yang H. (2017). Disrupted brain connectivity networks in acute ischemic stroke patients. Brain Imaging Behav..

[b0695] Zhu D., Chang J., Freeman S., Tan Z., Xiao J., Gao Y., Kong J. (2014). Changes of functional connectivity in the left frontoparietal network following aphasic stroke. Front. Behav. Neurosci..

